# Genome-Wide Association Study of Hepatocellular Carcinoma in Southern Chinese Patients with Chronic Hepatitis B Virus Infection

**DOI:** 10.1371/journal.pone.0028798

**Published:** 2011-12-08

**Authors:** Kelvin Yuen-Kwong Chan, Chun-Ming Wong, Johnny Sheung-Him Kwan, Joyce Man-Fong Lee, Ka Wai Cheung, Man Fung Yuen, Ching Lung Lai, Ronnie Tung-Ping Poon, Pak Chung Sham, Irene Oi-Lin Ng

**Affiliations:** 1 State Key Laboratory for Liver Research, The University of Hong Kong, Hong Kong; 2 Department of Pathology, The University of Hong Kong, Hong Kong; 3 Department of Psychiatry, The University of Hong Kong, Hong Kong; 4 Department of Medicine, The University of Hong Kong, Hong Kong; 5 Department of Surgery, The University of Hong Kong, Hong Kong; Ohio State University Medical Center, United States of America

## Abstract

One of the most relevant risk factors for hepatocellular carcinoma (HCC) development is chronic hepatitis B virus (HBV) infection, but only a fraction of chronic HBV carriers develop HCC, indicating that complex interactions among viral, environmental and genetic factors lead to HCC in HBV-infected patients. So far, host genetic factors have incompletely been characterized. Therefore, we performed a genome-wide association (GWA) study in a Southern Chinese cohort consisting of 95 HBV-infected HCC patients (cases) and 97 HBV-infected patients without HCC (controls) using the Illumina Human610-Quad BeadChips. The top single nucleotide polymorphisms (SNPs) were then validated in an independent cohort of 500 cases and 728 controls. 4 SNPs (rs12682266, rs7821974, rs2275959, rs1573266) at chromosome 8p12 showed consistent association in both the GWA and replication phases (OR_combined_ = 1.31–1.39; p_combined_ = 2.71×10^−5^–5.19×10^−4^; PAR_combined_ = 26–31%). We found a 2.3-kb expressed sequence tag (EST) in the region using *in-silico* data mining and verified the existence of the full-length EST experimentally. The expression level of the EST was significantly reduced in human HCC tumors in comparison to the corresponding non-tumorous liver tissues (*P*<0.001). Results from sequence analysis and in-vitro protein translation study suggest that the transcript might function as a long non-coding RNA. In summary, our study suggests that variations at chromosome 8p12 may promote HCC in patients with HBV. Further functional studies of this region may help understand HBV-associated hepatocarcinogenesis.

## Introduction

Approximately 400 million persons worldwide are chronically infected with hepatitis B virus (HBV) [1], [2]. Carriers of HBV are at an increased risk of developing cirrhosis, hepatic decompensation, and hepatocellular carcinoma (HCC) development, especially in the endemic areas of Southeast Asia, China, and Sub-Saharan Africa. The virus is one of the most relevant risk factors for HCC. However, only a fraction (incidence rate between 33.5 and 2,632 per 100,000 person-years for men in different populations) of chronic HBV carriers develops HCC. This indicates that complex interactions among viral, environmental and genetic factors lead to HCC in HBV-infected patients. So far, host genetic factors have incompletely been characterized and only a few studies identified genes potentially conferring genetic susceptibility to HBV-associated HCC [3]–[9].

Genome-wide association (GWA) study has greatly contributed to the identification of common genetic variants related to common diseases [10]. This hypothesis-free approach allows the discovery of novel genetic loci previously not thought to be associated with the disease. A recent GWA study has reported a susceptibility locus for HBV-related HCC at 1p36.22; it has shown that genes situated at the proximal region were aberrantly expressed in HCC tumors [11]. Since population substructure exists among Chinese living in different geographical regions [12], the use of a more homogeneous population may improve power to detect new risk loci. Therefore, we performed a GWA study in a Southern Chinese cohort consisting of 95 HBV-infected HCC patients (cases) and 97 HBV-infected patients without HCC (controls) using the Illumina Human610-Quad BeadChips. The top single nucleotide polymorphisms (SNPs) were then validated in an independent cohort of 500 cases and 728 controls. Four SNPs (rs12682266, rs7821974, rs2275959, rs1573266) at chromosome 8p12 showed consistent association in both the GWA and replication phases (OR_combined_ = 1.31–1.39; p_combined_ = 2.71×10^−5^–5.19×10^−4^; population attributable risk (PAR)_combined_ = 26–31%). Using *in-silico* data mining, we found no gene but a 2.3 kb expressed sequence tag (EST) in the 8p12 region. Expression analysis of the EST in HCC and adjacent non-tumorous liver tissues was performed and in-vitro transcription and translation analysis of the EST were also done to examine the EST's involvement in HCC development.

## Materials and Methods

### Patient Selection and Materials

All cases and controls were recruited at Queen Mary Hospital, University of Hong Kong, and were ethnically Southern Chinese. All cases were positive for the hepatitis B surface antigen (HBsAg) and were examined physically for symptoms and signs of HCC and cirrhosis. Progressed carriers with HCC were defined as HBsAg carriers with a serum alpha-fetoprotein level above 400 ng/mL and typical computed tomography findings. Histological examinations using fine-needle aspiration performed under ultrasonographic guidance were also used to define HCC tumors. Informed signed consent was obtained from the study subjects prior to peripheral blood collection. Ethical approval was obtained from the Institutional Review Board.

Ninety-five cases and 97 age- and sex-matched controls were recruited for genome-wide genotyping in 2007. Each control was 5 years older than the age of onset of his/her matched case to ensure that he/she was not developing HCC in the past 5 years. The mean ± SD onset age of cases, defined as the age at first diagnosis of HCC, was 50.6±7.3 years, while the mean ± SD age of controls was 55.6±7.3 years. Further recruitment of cases and controls was performed in 2008–2010, resulting in a total of 500 cases and 728 controls for independent validation. The mean ± SD onset age of these cases was 52.8±9.0 years and the mean ± SD age of the controls was 57.6±10.5 years. The male to female ratio in both cases and controls were about 4∶1. In the genome-wide genotyping cohort, 13 (13.7%) of the cases and 3 (3.1%) of the controls had cirrhosis; only one case and none of the controls had HBV/HCV co-infection; 11 (11.6%) of the cases and one of the controls had alcohol consumption more than 60 g per day; none of the cases and 16 (16.5%) of the controls had antiviral treatment (Lamivudine, Adefovir and both).

### DNA Extraction

Peripheral blood samples collected in EDTA blood tubes were processed for DNA extraction on the day of collection. QIAmp Blood DNA Midi and Mini kits (Qiagen, Valencia, CA, USA) were used as described in the manufacturer's protocol. DNA was verified by gel electrophoresis; DNA purity was assessed by OD 260/280 ratio; DNA concentration was determined by the Quant-iT™ PicoGreen® dsDNA reagent (Life Tech., Carlsbad, CA, USA).

### Genotyping and Statistical Analysis

Genome-wide genotyping was carried out at deCODE Genetics (Reykjavik, Iceland) using the Illumina Human610-Quad BeadChips. We excluded related samples by allele sharing analysis using PLINK v1.05 [13], outlier samples by principal component analysis using EIGENSOFT v2.0 [14] and poor quality samples having call rates <95%. We also excluded SNPs with minor allele frequency (MAF) <0.01 in both cases and controls, having call rates <95% in both cases and controls, and having Hardy-Weinberg equilibrium *P-*value <0.001 in controls. After quality control, 192 samples (95 cases and 97 controls) and 485,072 SNPs remained for the subsequent association analyses.

Phenotype-genotype association for each SNP was assessed using logistic regression analysis adjusted for age and gender in PLINK. Population-attributable risk (PAR) was calculated as PAR = 1−{1/[(1−*p*)^2^+2*p*(1−*p*)*OR*+*p* ^2^ *OR* ^2^]}, where *p* is the risk allele frequency, assuming a multiplicative model. The most significant SNPs were genotyped in an independent sample of 500 cases and 728 controls using Sequenom (Sequenom Inc., San Diego, CA, USA) and were analyzed for association by chi-squared test. Association results from the GWA and replication studies were combined using inverse variance-weighted fixed effects meta-analysis. Since a previously reported susceptibility locus [11], rs17401966, was not present in the genotyping array, genotyping of this SNP was determined separately by TaqMan allelic discrimination assay (Life Tech) in the same cohort (357 cases and 354 controls) and was also analyzed for association by chi-squared test.

### 5′ and 3′RACE

5′ and 3′RACE were performed according to the manufacturer's instruction in the 5′/3′ RACE Kit 2^nd^ Generation (Roche Diagnostics, Mannheim, Germany). Total RNA was extracted from HepG2 cell lines using the Trizol reagent (Life Tech.). The extracted total RNA was subjected to *DNase* I treatment using the TURBO DNA-free kit (Applied Biosystems). For 5′RACE, the *DNase* I treated RNA was used as a template for first-strand cDNA synthesis with SP1 primer 5′-GAG GTG AAG ATC CTG TCA AAG GT-3′. The resulting products were then polyadenylated using dATP and were subsequently amplified using the Oligo(dT) primer and the SP2 primer 5′-GAA CGC ACC AGA TAA GAT CTG AG-3′. Similar to 5′RACE, the first-strand cDNA synthesis in 3′RACE was performed using the Oligo(dT)-anchor primer (provided in the kit). The subsequent PCR amplification was carried out using a PCR anchor primer (provided in the kit) and the SP5 primer 5′-AAT AGC TTA ACC CTT TCA TTT ACC A-3′. The resulting products from 5′ and 3′RACE were cloned into a pGEM-T Easy Vector (Promega, Madison, WI, USA) and verified using DNA sequencing.

### Quantitative PCR (qPCR) and Quantitative Expression Analysis

The expression level of the EST transcript was examined in 40 pairs of HCC tumors and adjacent non-tumorous tissues using relative qPCR with transcript specific primers as shown below. Total RNA was extracted from the tissues using Trizol reagent (Life Tech.). Complementary DNA was synthesized from 1 µg of total RNAs using MultiScribe™ Reverse Transcriptase and Oligo(dT) (Life Tech.) in which total RNA was treated with TURBO DNA-free to remove possible DNA contamination. Each qPCR reaction consisted of a 1× FastStart SYBR Green I reaction mixture (Roche Diagnostics), 500 nM of each forward (CTC AGA TCT TAT CTG GTG CGT TC) and reverse (GAG GTG AAG ATC CTG TCA AAG GT) primer, and 1 µL of synthesized cDNA. The expression of a housekeeping gene of *GAPDH* was examined and used for normalizing the expression of the EST wild-type transcript. The expression level of the EST between the HCC tumors and corresponding non-tumorous liver tissues was examined using a non-parametric paired *t*-test, in which *P*<0.05 was considered significant.

### Translation of FLAG-tagged EST Open Reading Fframe (ORF)

The predicted largest ORF (encoding 94 amino acids) of the EST transcript was amplified using the following sets of primers: for N-terminal FLAG-tagged EST ORF, forward 5′- CGC GAA TTC ATG GAC TAC AAA GAC GAT GAC GAC AAG CTT ATG AAA TAT AAT CAA GCA ATT AAC-3′ and reverse 5′- CGC GGA TCC GAG AGA GGT GAA GAT CCT GTC AAA G-3′; for C-terminal FLAG-tagged EST ORF, forward 5′- CGC GAA TTC ATG AAA TAT AAT CAA GCA ATT AAC-3′ and reverse 5′- CGC GGA TCC TCA AAG CTT GTC GTC ATC GTC TTT GTA GTC GAG AGA GGT GAA GAT CCT GTC AAA G-3′. The respective resulting amplicons were cloned into a lentiviral expression vector, pCDH-CMV-EF1-GFP-Puro (SBI System Biosciences, Mountain View, CA, USA) at the cloning sites of EcoRI and BamHI. The N- and C-terminal FLAG-tagged lentiviral expression plasmids were packaged into the lentiviruses which were then transduced to the HepG2 cell line as described previously [15]. Mock transduction using the empty vector was run in parallel. The transduced HepG2 cells were lysed in NET Buffer. The lysates were then resolved via 12% SDS-PAGE gel electrophoresis and blotted onto nitrocellulose membrane. The membrane was incubated with mouse anti-FLAG primary antibody, followed by incubation with anti-mouse immunoglobulin G (GE Healthcare, Buckinghamshire, UK), and was then detected using ECL plus Western blotting detection system (GE Healthcare) according to the manufacturer's protocol. Detection of endogenous β-actin was performed as for protein loading control.

## Results

### GWA and replication studies suggest that the 8p12 region contains susceptibility locus for HBV-related HCC

**Figure 1:**
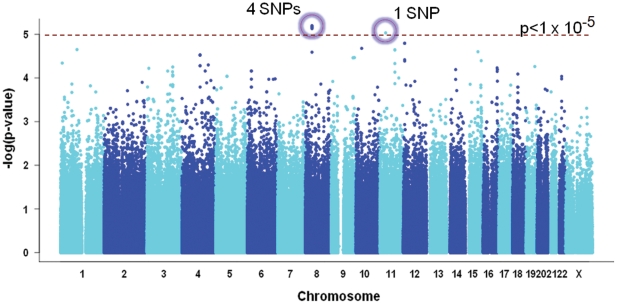
Manhattan plot of GWA study results of testing for association with HBV-related HCC susceptibility. The dotted line indicates the p-value threshold of 1×10^−5^. The circled SNPs are those passing the threshold (Table 1).

In the GWA study, there was no SNP reaching genome wide significance (p = 5×10^−8^). As shown in Figure 1, the strongest association signal was seen in the intergenic region at chromosome 8 (8p12; p = 6.35×10^−6^) driven by rs2275959. The association signal from this SNP was supported by associations from nearby SNPs (rs12682266, rs7821974, rs1573266) in linkage disequilibrium (LD) (Table 1), indicating that the signal was likely to be real. In contrast, the next strongest signal located on chromosome 11p13 (rs2611145, p = 9.31×10^−6^) had no other SNP in the region which show similar levels of association, indicating that it was likely to be a false positive result. We then genotyped the 4 SNPs in the 8p12 region in an independent sample (500 cases and 728 controls). The risk alleles of these SNPs were the same as in the GWA study (Table 1), but the combined analysis of the GWA study and replication samples revealed only suggestive associations at these 4 SNPs (OR_combined_ = 1.31–1.39; p_combined_ = 2.71×10^−5^–5.19×10^−4^; PAR_combined_ = 26–31%).

**Table 1:** Summary of association of SNPs in GWA and replication studies.

Chr	SNP	Position^a^	Risk/non-risk allele	GWA study (N_case_ = 95, N_control_ = 97)				Replication (N_case_ = 500, N_control_ = 728)				Combined (N_case_ = 595, N_control_ = 825)				
				RAF in cases	RAF in controls	OR	P-value	RAF in cases	RAF in controls	OR	P-value	RAF in cases	RAF in controls	OR	P-value	PAR
8	rs12682266	37548149	G/A	0.64	0.41	2.55	6.69×10^−6^	0.55	0.49	1.25	7.10×10^−3^	0.56	0.48	1.38	3.76×10^−5^	0.285
8	rs7821974	37569159	T/C	0.62	0.39	2.54	7.01×10^−6^	0.54	0.49	1.20	2.61×10^−2^	0.55	0.48	1.33	2.32×10^−4^	0.255
8	rs2275959	37574217	T/C	0.54	0.31	2.59	6.35×10^−6^	0.46	0.42	1.18	4.75×10^−2^	0.47	0.41	1.31	5.19×10^−4^	0.213
8	rs1573266	37581577	C/T	0.69	0.47	2.58	7.44×10^−6^	0.59	0.54	1.26	5.20×10^−3^	0.61	0.53	1.39	2.71×10^−5^	0.313
11	rs2611145	34240437	T/C	0.52	0.29	2.56	9.31×10^−6^	-	-	-	-	-	-	-	-	-

Chr, chromosome; RAF, risk allele frequency; OR, odds ratio.

a Based on NCBI build 36.

### The SNP rs17401996 was not associated with HBV-related HCC susceptibility

To test whether a previously reported susceptibility locus [11], rs17401996, was associated with HBV-related HCC susceptibility in our samples, we also genotyped the SNP in 357 HBV-positive HCC cases and 354 HBV-positive non-HCC controls using TaqMan allelic discrimination assay. We found no significant difference (p = 0.91) in allele frequencies between cases and controls (71.1% in cases versus 71.4% in controls).

### *In Silico* Data Mining Identified EST at 8p12

Results of *in silico* data mining (Figure S1) showed that there are no genes in the 8p12 region; however, an EST was identified within the studied haplotype block and encompasses the rs2275959 and rs2298321 SNPs, which were located in the HCC risk-associated haplotype block found in GWA. More notably, this EST was expressed in the HepG2 hepatoma cell line, and it was also detected in other human tissue types, such as prostate, intestine, placenta, and testis, according to the Stanford SOURCE database. The results of an ENCODE Histone Modification ChIP-Seq study on a panel of human cell lines (Figure S1) revealed that the chromatin region harboring the EST, particularly in the HepG2 cell line, had a remarkably high level of histone 3 lysine-4 methylation and acetylation as compared to the other examined cell lines. This result indicates that the EST may be actively expressed in this cell line. In fact, the expression of this transcript was detected by RT-PCR in various HCC cell lines, including HepG2, HLE, Huh7, BEL-7402, and PLC/PRF/5 (data not shown).

### Sequence and Expression Analysis of the EST at 8p12

**Figure 2:**
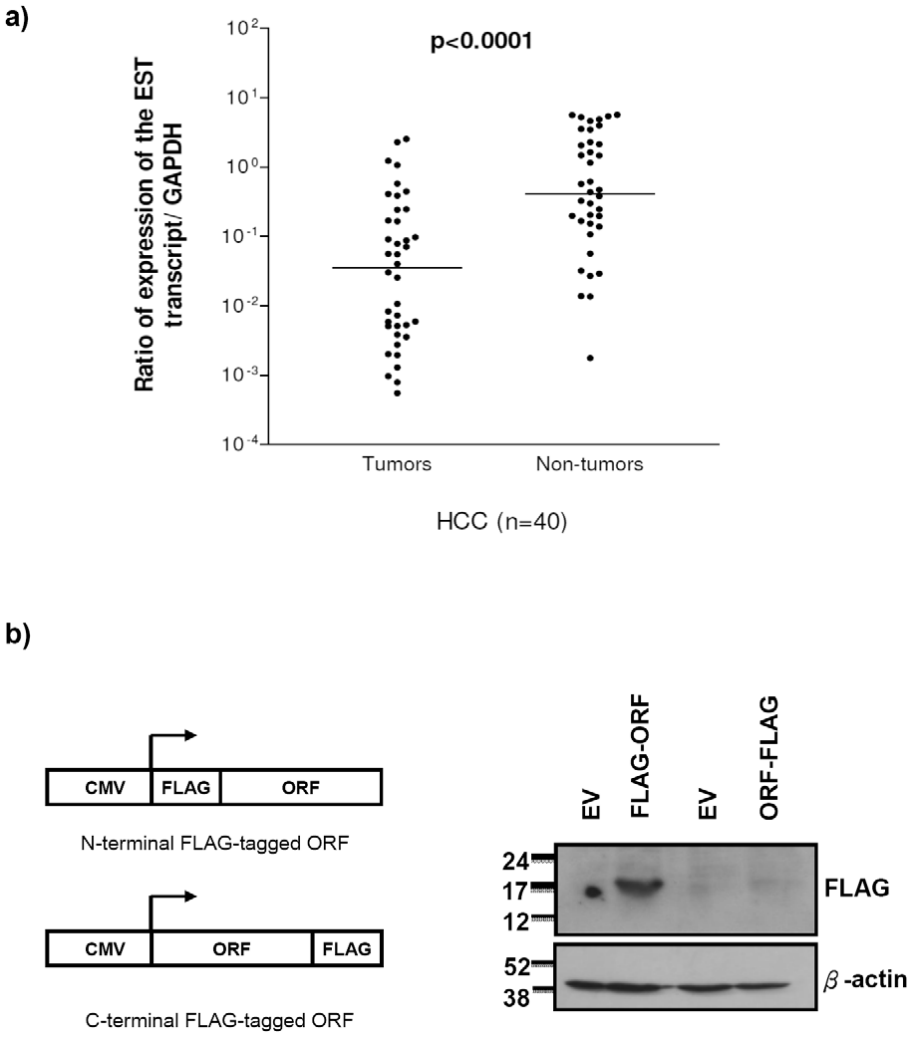
Expression of the EST in human HCCs and cell line. (A) Quantitative expression analysis of the EST transcript in the HBV-associated HCCs showed that the EST transcript was significantly under-expressed in HCC tumors when compared with the corresponding non-tumorous livers (*P*<0.0001, non-parametric paired *t*-test). Dots: the ratio of expression level of the transcript versus the level of GAPDH in each testing sample. Horizontal lines: mean of the relative expression level in the HCC tumors and non-tumorous livers. (B) Left panel: Schematic diagram of the FLAG-tagged expression constructs. Right panel: Western blot analysis of FLAG-tagged EST translation in HepG2 cell line. The FLAG-tag could be translated only when tagged at the N-terminus of the ORF but not at the C-terminus. β-actin served as a loading control. EV: empty vector, FLAG-ORF: N-terminal FLAG-tagged ORF, ORF-FLAG: C-terminal FLAG-tagged ORF.

We confirmed the full-length sequence of the EST transcript (2.3 kb) and demonstrated that the 3′ end of the transcript was polyadenylated by 5′/3′RACE. The 2.3 kb transcript is intronless as reported (GenBank:AK025743). Primers designed to specifically detect the transcript were used to quantify the relative expression level of this transcript and revealed that its expression level was significantly reduced in HBV-associated HCC tumors, as compared to the corresponding non-tumorous liver tissues (*P*<0.001, Figure 2A). Based on the possibility that the transcript could encode a protein, we performed a sequence analysis of the hypothetical protein product of the transcript, LOC157860, using InterProScan, which indicates that the protein sequence possesses a signal-peptide sequence and a transmembrane region in the N-terminal half of the sequence. Despite the 2.3 kb length of the transcript, only very short open reading frames (ORF) were predicted. The largest ORF encodes only 94 amino acids while the three other predicted ORFs encode 54, 55, and 67 amino acids, respectively. We then stably transduced HepG2 cells with expression plasmids containing either N-terminal or C-terminal FLAG-tagged ORF (encoding 94 amino acids) (Figure 2B). The C-terminal FLAG-tagged EST ORF was detected by anti-FLAG immunoblotting in the transduced HepG2 cell lysate and had the expected size in the immunoblotting results, but the N-terminal FLAG-tagged one was not translated in its corresponding cell lysate (Figure 2B). We therefore propose that the transcript might act as a large intervening ncRNA, but further investigation is needed.

## Discussion

In this study, we identified a new candidate susceptibility locus on chromosome 8p12 for HBV-associated HCC in Southern Chinese using GWA study. *In-silico* data mining showed that an EST and its transcript isoforms are situated in this region. The full length of the transcript was verified by 5′and 3′RACE and DNA sequencing. The region contained high levels of chromatin activation marks (UCSC Browser), which was additionally observed in the hepatoma cell line HepG2, indicating transcriptional activation. A comparative genomic analysis indicates that the transcript region is evolutionarily conserved in mammals (Figure S2) and suggests that this region may be of some functional importance.

Previous reports investigating the loss of heterozygosity and comparative genomic hybridization have shown that region 8p was one of the most frequently deleted regions in HCCs, as demonstrated by our previous study [16] and other studies [17]. Notably, the well-known liver cancer suppressor gene, Deleted in Liver Cancer 1 (DLC1),[18] is also situated on chromosome arm 8p. It spans over the region of p21.3–22 and is about 24 Mb upstream of the four 8p12 SNPs. We speculate that risk-associated 8p12 SNPs or haplotypes might have an interacting effect on the DLC1 locus, causing DLC1 more susceptible to deletion or chromosomal loss; however, the underlying mechanism of such effect remains to be investigated.

In this study, we confirmed the full-length sequence of the EST transcript (2.3 kb) and demonstrated that the 3′ end of the transcript was polyadenylated by 5′/3′RACE. The expression level of this transcript was significantly reduced in HBV-associated HCC tumors, as compared to non-tumorous liver tissues. Significantly, the transcript was also detected in other HCC cell lines, specifically HLE, Huh7, BEL-7402, and PLC/PRF/5. Despite the 2.3 kb length of the transcript, only very short ORFs were predicted, with the largest encoding only 94 amino acids. The C-terminal FLAG-tagged ORF was detected by anti-FLAG immunoblotting in the transduced HepG2 clones, but its N-terminal FLAG-tagged one was not expressed. The evidence is consistent with the characteristics of ncRNAs suggested by Panzitt et al.[19] We therefore propose that the transcript might act as long ncRNAs, but further investigation is required. Moreover, it remains to be investigated whether the reduction in expression is limited to HCCs. ncRNAs are a class of molecules that play regulatory roles in cells. Notably, several ncRNAs are implicated in human cancers. In contrast to proteins that are encoded by coding RNAs, the function of ncRNAs cannot currently be inferred from their sequence or structure. A large intervening ncRNA (lincRNA), which can range from 200 nt to >100 kb, can be intergenic, intronic, antisense or overlapping with protein-coding genes or other ncRNAs [20], [21]. The known repertoire of lincRNA functions is still being unveiled with roles as mediator of mRNA decay [22], structural scaffolds for nuclear substructures [23], and as regulators of chromatin remodeling [24]. For instance, the HOTAIR lincRNA was reported to be overexpressed in breast tumors and found to be a significant predictor of metastasis and death [25]. Functional analyses revealed that HOTAIR silences the *HOXD* locus *in trans* by inducing a repressive chromatin state. Similarly, overexpression of the nuclear speckle associated lincRNA metastasis-associated lung adenocarcinoma transcript 1 (*MALAT1*) modulates alternative splicing. It was found associated with metastasis and poor outcome in patients with lung cancer [26]. Another recent report also identified and demonstrated that the HULC long ncRNA is differentially expressed in HCC tumors [19].

To the best of our knowledge, this study is the second largest GWA study of HBV-related HCC susceptibility in Chinese and the largest in Southern Chinese, but no SNP achieved genome-wide significance (p<5×10^−8^). Power should be an important factor that influences the outcome of our current study. Our GWA study and combined samples had 0.004% and 6.65% power, respectively, for an OR of 1.35, assuming a disease prevalence of 0.65% and a 50% MAF. It is estimated that at least 1,700 cases and an equal number of controls are required to achieve a power of 80%. So, increasing sample size is one of the options to greatly improve the power of our study. However, by adopting a less stringent significant threshold of *P*<1×10^−5^ which had recently been used in another large scale GWA study [27], and after demonstrating the consistent association in the validation sample set and illustrating the in-vivo finding of reduction of the 8p12 EST expression in HCC, we speculated the 8p12 region is a new HBV-associated HCC susceptible locus. Nevertheless, we could not exclude the possibility that additional common genetic variants with similar effect to those 8p12 SNPs exist.

In our study population, a previously reported susceptibility locus at 1p36.22 [11], rs17401966, was not significantly associated with HBV-related HCC. We had 80%, 60% and 34% power, respectively, for an OR of 1.4, 1.3 and 1.2, assuming a disease prevalence of 0.65% and a 30% MAF. So, sample size is unlikely to be the major drawback for studying the rs17401966 SNP in our cohorts. There might be some other confounding genetic factors, which are different from our Southern Chinese cohorts, interplaying with the risk associated 1p36.22 locus, hence contributing to the development of HCC. It has recently demonstrated that Han Chinese populations indeed exit of different genetic structures [12]. Comparing the Guangdong cohorts used in Zhang et al and our cohorts genotyped for the rs17401966 SNP, there is a great evidence of heterogeneity with P_het_ = 0.035 by test of heterogeneity (Chi-square, 1df, 2-tailed) although Hong Kong is situated closed to the Guangdong province. The degree of heterogeneity is even greater when comparing ours to the Northern Chinese Beijing cohorts (P_het_ = 0.0017). Owing to these genetic difference among Chinese populations, originality of patients and the genetic components, apart from those non-genetic factors, can be used for HCC risk assessment and disease management. The joint effect of genes in contributing to cancer risk supports polygenic model. Polygenic risk score assessed by providing genotyping results of some genetic variants/SNPs has been reported to provide better risk prediction for other hunan cancers, such as prostate [28] and breast cancers [29].

Overall, we identified a HBV-associated HCC susceptibility locus, which harbors an EST whose expression is significantly associated with HBV-associated HCC development. Further validation using a larger sample size and/or multiple populations, particularly in Chinese patients, is helpful to substantiate our findings. The functions of this EST transcript should also be investigated.

## Supporting Information

Figure S1 **Computational data retrieval from the UCSC Genome browser database on the human 8p12 region (chr8:37,560,000–37,600,000 on Human NCBI36/hg18 assembly).** The location of the EST transcript (GeneBank: AK025743) is indicated by the black bar (chr8:37,574,170–37,576,534). The histone 3 methylation and acetylation marks (H3K4me1, H3K27Ac, and H3K4me3) are indicated by the colored peaks; each color represents the results of one type of human cell line, such as H1 ES, HMEC, HSMM, HUVEC, K562, NHEK, NHLF, and HepG2. The H3K27Ac and H3K4Me3 marks are particularly high in HepG2, as indicated by the light green peaks.
(TIF)

Figure S2 **DNA conserved region analysis in mammals.** Conserved regions are highlighted with shaded colored bars.
(TIF)
