# Supplementary figures and images for: Genome-Wide Association Study of Hepatocellular Carcinoma in Southern Chinese Patients with Chronic Hepatitis B Virus Infection
Source: PLoS One. 2011 Dec 8;6(12):e28798. doi: 10.1371/journal.pone.0028798 (PMC3234276; doi:10.1371/journal.pone.0028798)

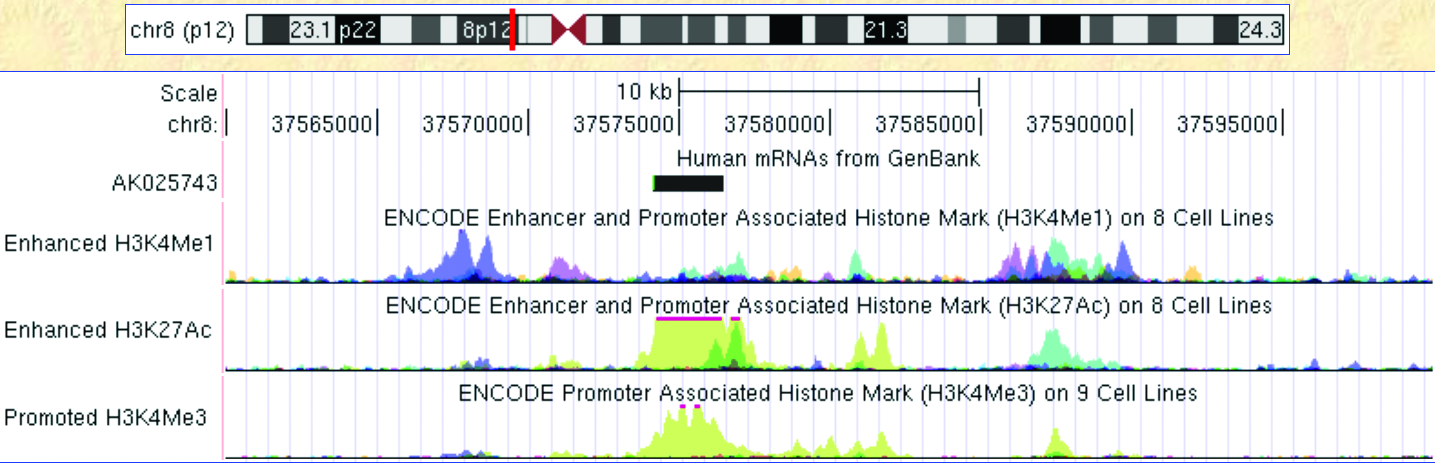

Supplement: Figure S1 — Computational data retrieval from the UCSC Genome browser database on the human 8p12 region (chr8:37,560,000–37,600,000 on Human NCBI36/hg18 assembly). The location of the EST transcript (GeneBank: AK025743) is indicated by the black bar (chr8:37,574,170–37,576,534). The histone 3 methylation and acetylation marks (H3K4me1, H3K27Ac, and H3K4me3) are indicated by the colored peaks; each color represents the results of one type of human cell line, such as H1 ES, HMEC, HSMM, HUVEC, K562, NHEK, NHLF, and HepG2. The H3K27Ac and H3K4Me3 marks are particularly high in HepG2, as indicated by the light green peaks. (TIF) [file pone.0028798.s001.tif]

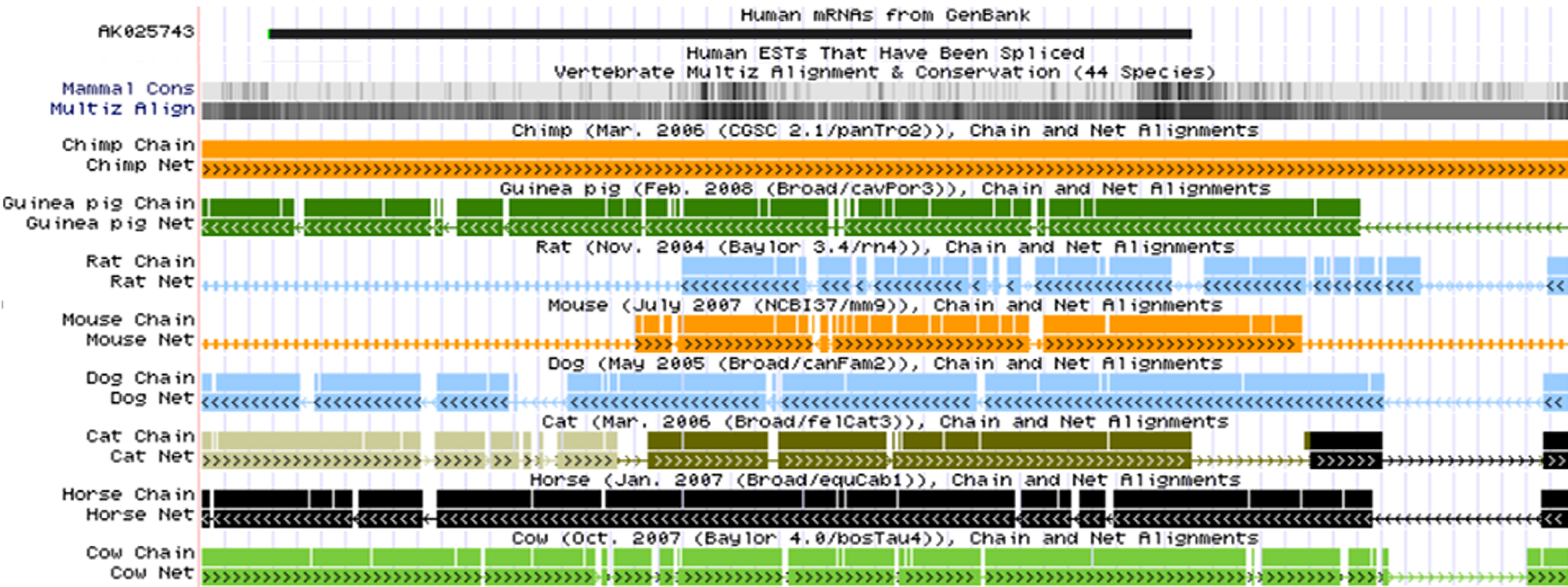

Supplement: Figure S2 — DNA conserved region analysis in mammals. Conserved regions are highlighted with shaded colored bars. (TIF) [file pone.0028798.s002.tif]
